# A rare case of Gorham-stout syndrome involving the thoracic spine with progressive bilateral chylothorax: a case report

**DOI:** 10.1186/s12891-019-2542-z

**Published:** 2019-04-09

**Authors:** Peng Wang, Wenbo Liao, Guangru Cao, Yongyan Jiang

**Affiliations:** 1 The Affiliated Hospital of Zunyi Medical University, Zunyi, China; 2 The Third Affiliated Hospital of Zunyi Medical University, Zunyi, China

**Keywords:** Gorham-stout syndrome, Thoracic spine, Chylothorax

## Abstract

**Background:**

Gorham-Stout syndrome (GSS) with spinal involvement is extremely rare, and bilateral chylothorax as a complication is usually fatal. In our case, pleural effusion appeared in the left hemithorax after ligating the right thoracic duct.

**Case presentation:**

A 14-year-old patient presented with GSS affecting the thoracic spine with bilateral chylothorax. The case was successfully managed using combined conservative and surgical treatments. At the 2-year follow-up visit, the amount of pleural fluid was reduced, the patient’s respiratory function had improved, and the deformity on the thoracic spine had gradually stabilized.

**Conclusions:**

GSS is a rare disorder of the musculoskeletal system that responds poorly to therapies and exhibits very high morbidity and mortality. Chylothorax is a common complication when lesions involve the thoracic spine, and physicians should be vigilant for possible serious pulmonary complications.

## Background

GSS is a bone disease of unknown etiology, characterized by idiopathic, progressive osteolysis and absorption with mono- or polyostotic lesions [1]. The main lesions are osteolysis and lymphatic tissue proliferation without malignancy [2]. GSS with spinal involvement is extremely rare, and its prognosis is poor [3]. In severe cases, visceral functions are affected, which may be life-threatening. One common complication is the development of chylothorax, which occurs in approximately 20% of patients and carries a high mortality rate [4]. No standard diagnosis or treatments exist for the disease, which is easily misdiagnosed. Here, we report the case of a 14-year-old girl who presented with GSS affecting the thoracic spine with progressive bilateral chylothorax, which was successfully managed with combined conservative and surgical treatments. We also describe the clinical manifestation, radiological features and histopathological characteristics in conjunction with a literature review.

## Case presentation

A previously healthy 14-year-old girl presented with cough, sputum and shortness of breath after activity. She had a history of trauma 10 days prior to presentation. She was previously admitted to another hospital and diagnosed with tuberculosis. Antituberculosis treatment was ineffective; therefore, she was transferred to our hospital. She had no family history of genetic or osteolytic disease. She was admitted to the respiratory department with dyspnea and persistent cough. Examination revealed tachypnea, diminished breathing sounds, a deformity on her back, and tenderness. She exhibited percussion pain in the T6–T9 vertebrae and an absence of motor power in the thoracic spine. Neurological examination was normal.

**Fig. 1:**
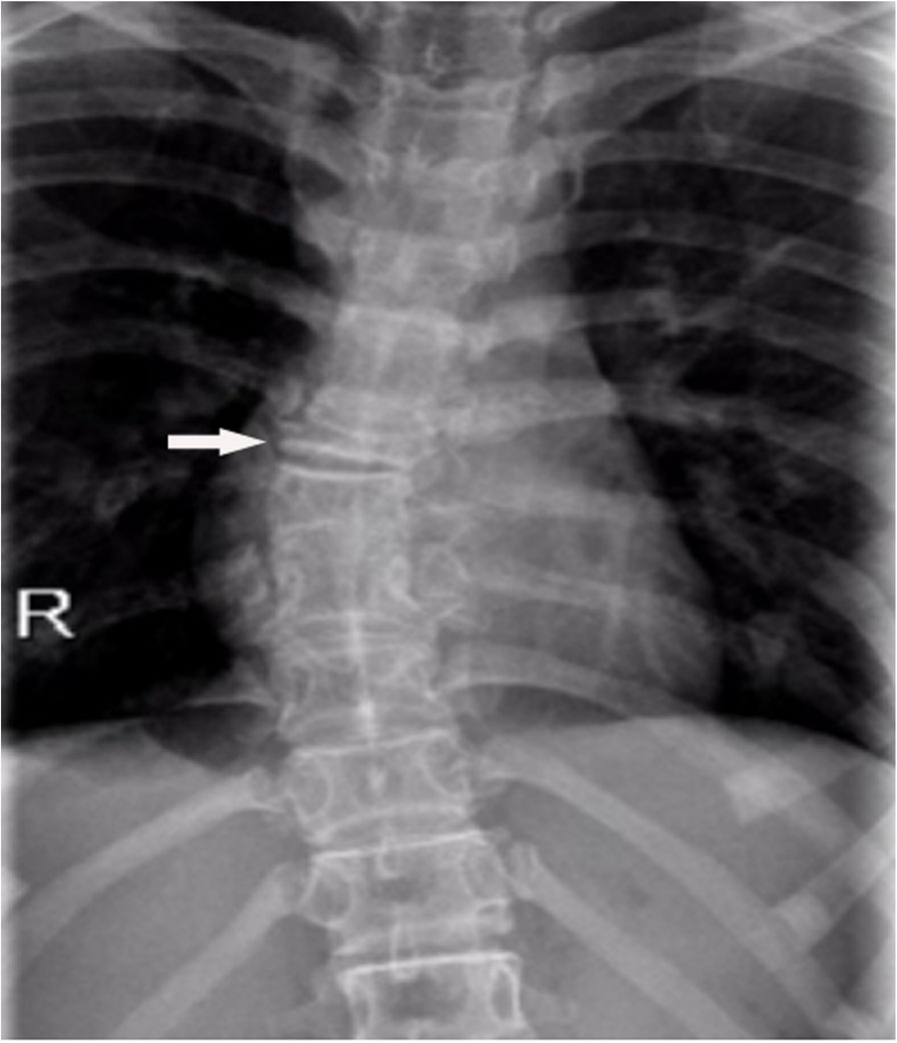
Radiographs. Multiple osteolytic lesions are visible in the ribs and thoracic vertebrae with scoliosis. (The white arrow indicates the destructed vertebrae)

**Fig. 2:**
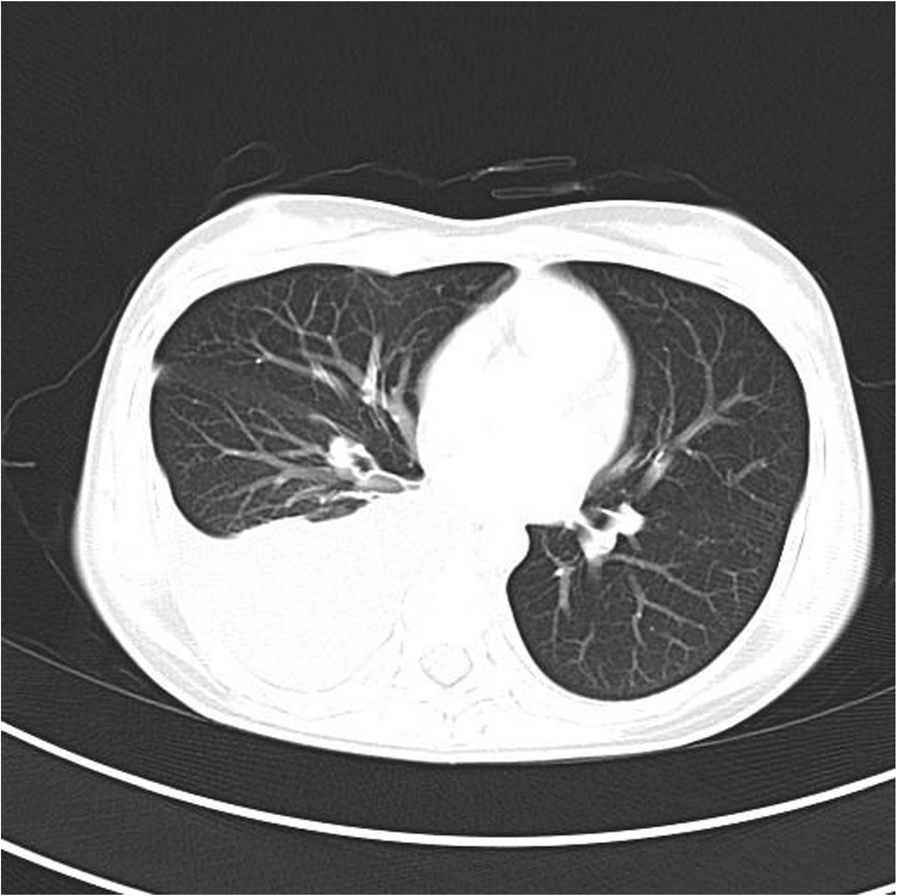
Chest CT. Pleural effusion and atelectasis are visible in the right hemithorax

**Fig. 3:**
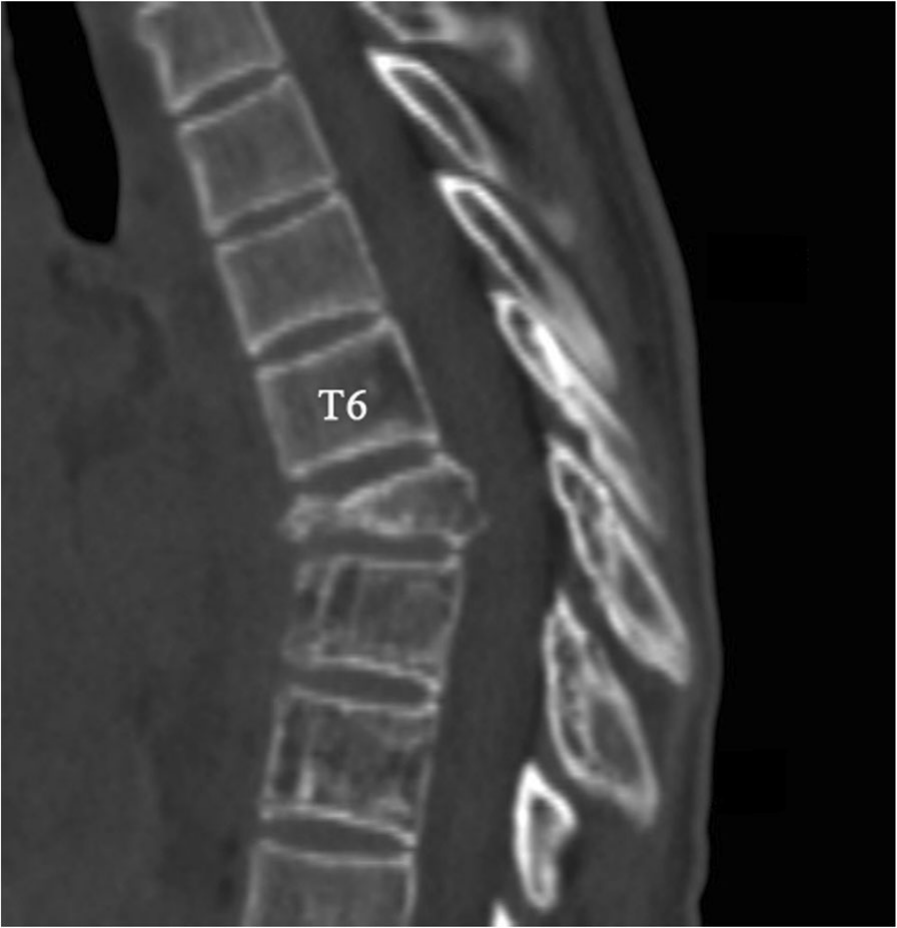
Thoracic CT. Multiple bone lesions are visible in thoracic vertebrae 6–9 with osteolysis of the ribs and a pathological fracture of the seventh thoracic vertebra. (The white arrow indicates the destructed vertebrae, the red arrow indicates the osteolytic rib, and the circle indicates pleural effusion)

**Fig. 4:**
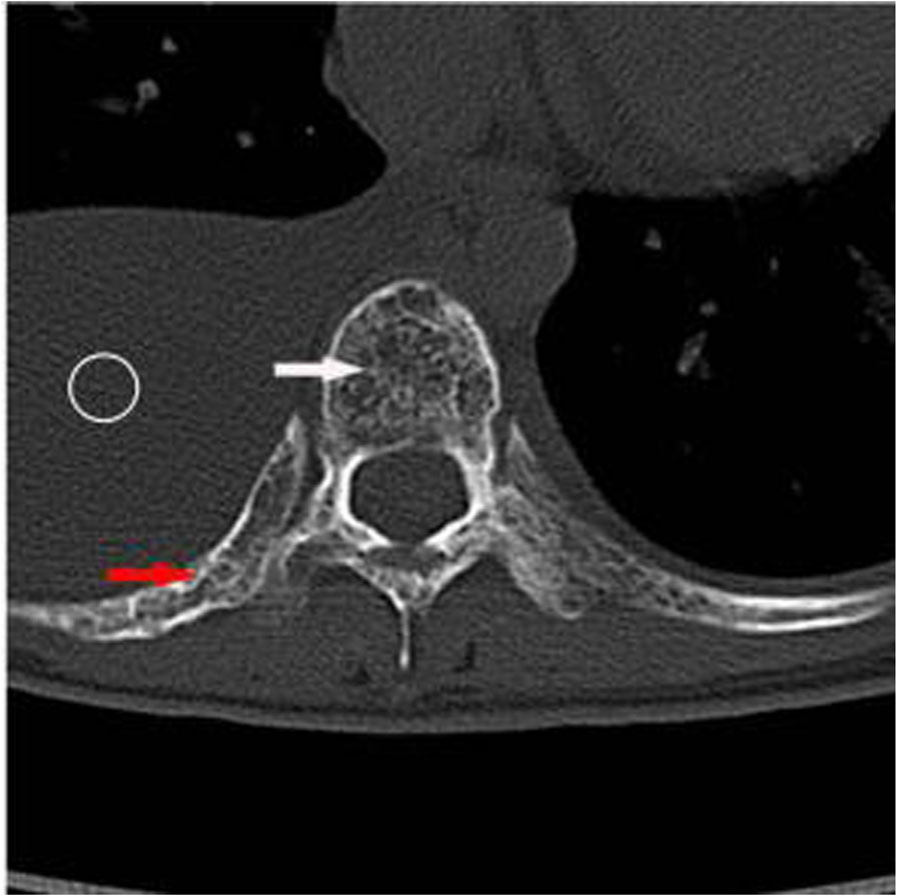
Thoracic CT. Multiple bone lesions are visible in thoracic vertebrae 6–9 with osteolysis of the ribs and a pathological fracture of the seventh thoracic vertebra. (The white arrow indicates the destructed vertebrae, the red arrow indicates the osteolytic rib, and the circle indicates pleural effusion)

**Fig. 5:**
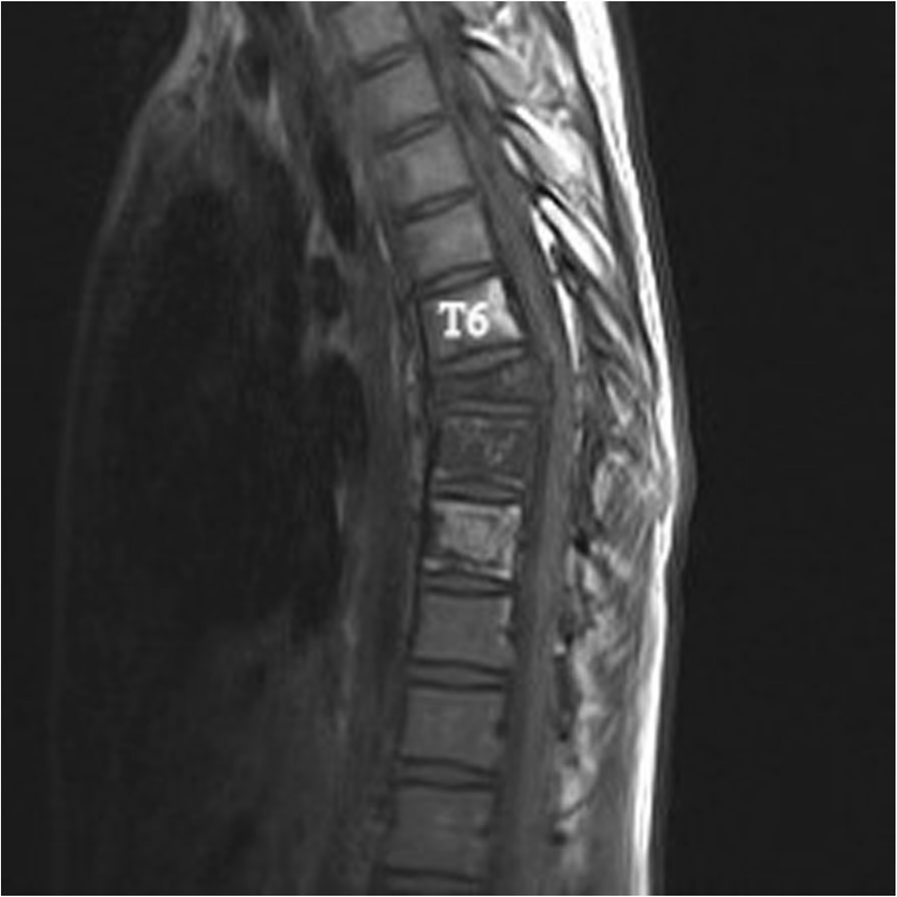
Magnetic resonance imaging (MRI). Sagittal plane of the MRI scan shows bony destruction of the T6–9 vertebrae combined with kyphosis and an unobstructed spinal canal

**Fig. 6:**
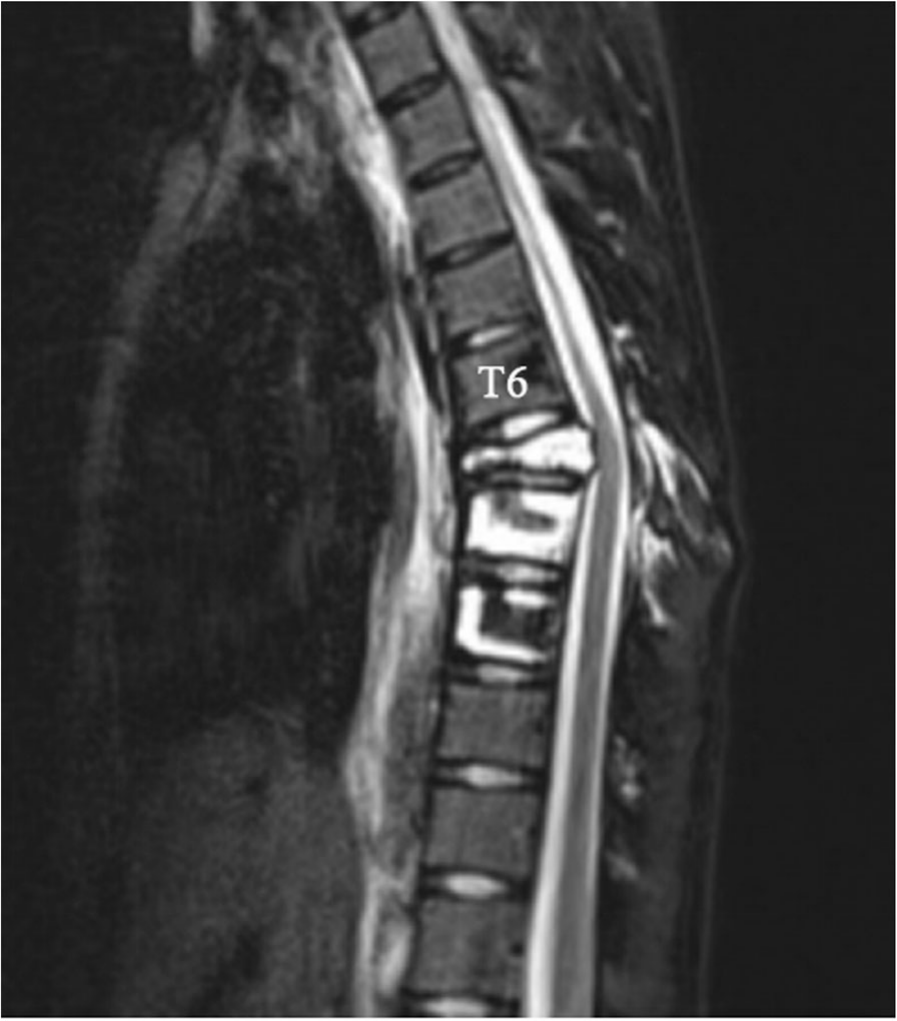
Magnetic resonance imaging (MRI). Sagittal plane of the MRI scan shows bony destruction of the T6–9 vertebrae combined with kyphosis and an unobstructed spinal canal

Plain radiographs revealed an osteolytic lesion in the thoracic spine (Fig. 1). Thoracic computed tomography (CT) showed a moderate right-sided pleural effusion and atelectasis (Fig. 2). Her thoracic spine CT revealed the presence of ill-defined lytic lesions in the ribs and the T6–T9 vertebrae as well as a fracture in the T7 vertebra (Figs. 3 and 4). Magnetic resonance imaging (MRI) scans revealed a pathological fracture and spinal canal stenosis at the T7 vertebra and high intensity in the T6–T9 vertebral bodies (Figs. 5 and 6). Whole-body bone scintigraphy was performed, and radiolucent foci were observed in the fracture lesion on the radiographic images. Blood analyses indicated nearly normal biochemical levels, except for a high concentration of cross-linked N-terminal telopeptides of type I collagen (111.60 ng/ml) and decreased vitamin D (8.99 ng/ml).

**Fig. 7:**
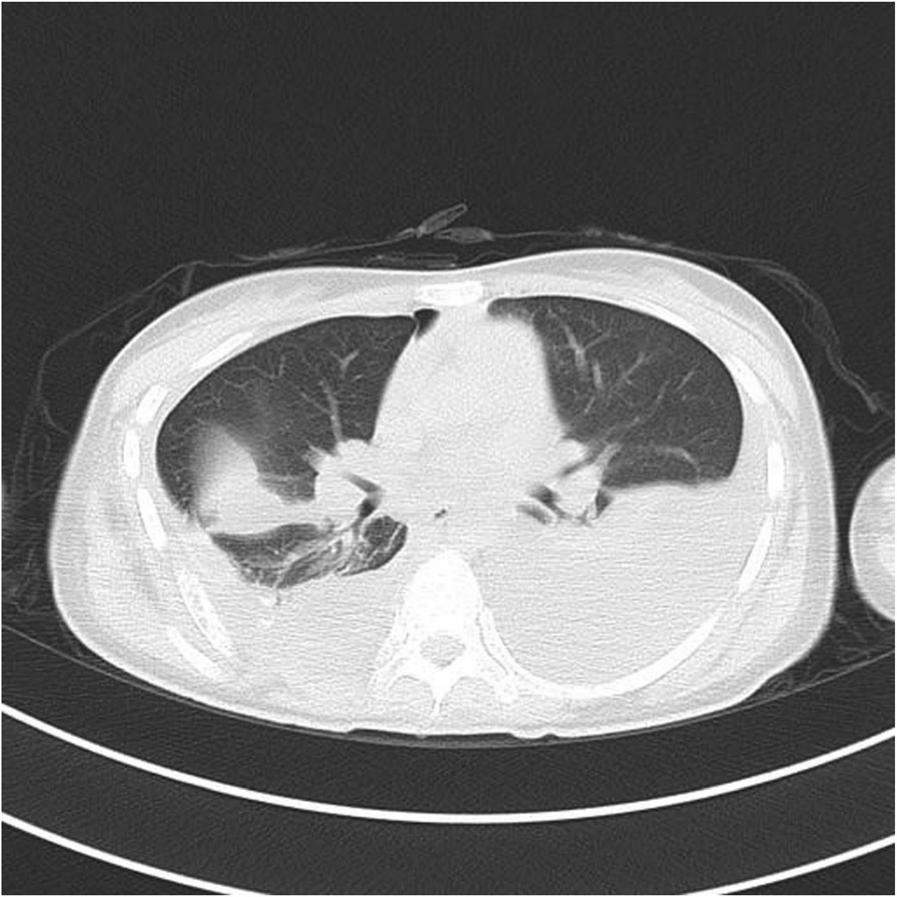
Chest CT after thoracic duct ligation. Bilateral pleural effusion is visible

**Fig. 8:**
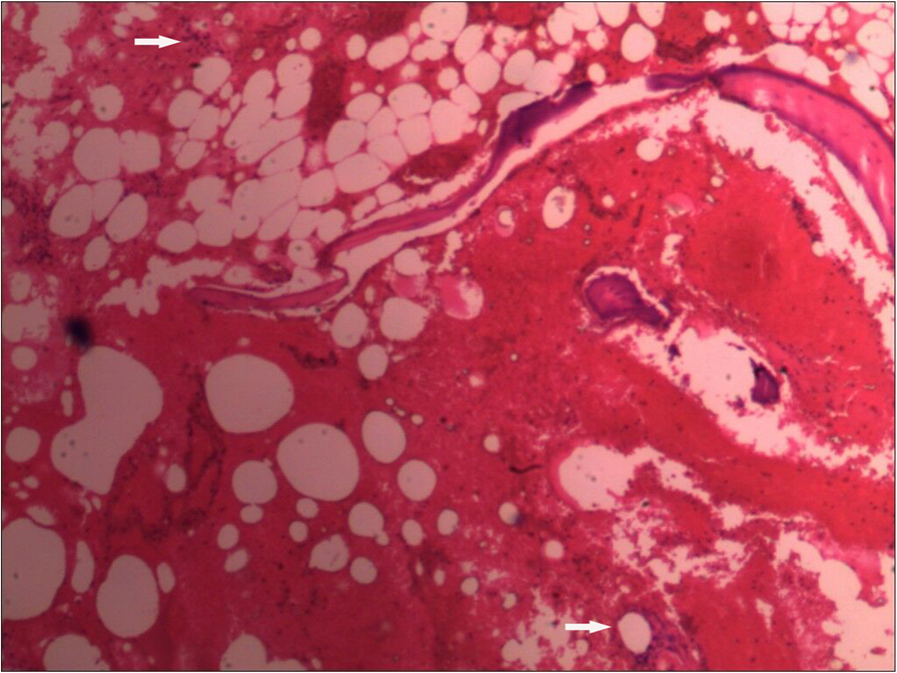
Pathological analyses. (Hematoxylin & eosin [H&E], 10× and 40×) More dilated sinusoids are visible with hemorrhaging, mononuclear cell and lymphocytic infiltration, fibrous tissue and dead bone. (The white arrow indicates dilated sinusoids and mononuclear cell and lymphocytic infiltration)

**Fig. 9:**
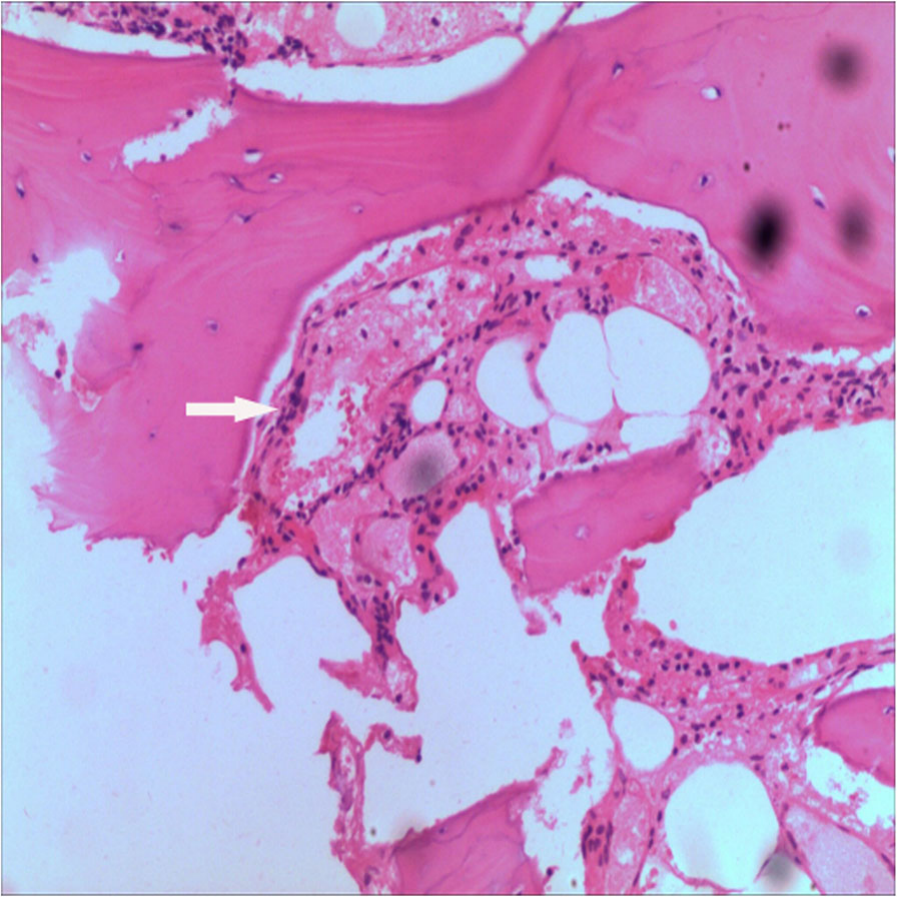
Pathological analyses. (Hematoxylin & eosin [H&E], 10× and 40×) More dilated sinusoids are visible with hemorrhaging, mononuclear cell and lymphocytic infiltration, fibrous tissue and dead bone. (The white arrow indicates dilated sinusoids and mononuclear cell and lymphocytic infiltration)

Recurrent chylothorax was managed via repeated thoracentesis, and percutaneous fine needle aspiration of the lesion yielded more than 1000 ml/day of a reddish turbid, nonodorous fluid. Analysis of the aspirate revealed a positive Rivalta test result, which was reported as chylothorax. The patient was transferred to the thoracic surgery department to control the pleural effusion. A thoracic duct ligation and pleurodesis along with chest drainage was planned. The biopsy could not be analyzed because insufficient tissue was taken from the lesion during the process. Chest CT showed bilateral pleural effusions 2 days after surgery (Fig. 7), and the chest was drained on the left side. To investigate the lesion pathology, the patient underwent another incisional biopsy of the T6–T9 vertebral bodies at the department of spine surgery. The bones appeared honeycomb-like intraoperatively. Postoperational pathological examination of the incisional biopsy revealed many dilated sinusoids with hemorrhaging, mononuclear and lymphocytic infiltration, fibrous tissue and dead bone (Figs. 8 and 9). Based on the clinical, radiological and pathological findings, we confirmed the diagnosis of GSS because the biopsy material was negative for bacterial and fungal growth, and osteolysis was clearly demonstrated in the imaging results.

**Fig. 10:**
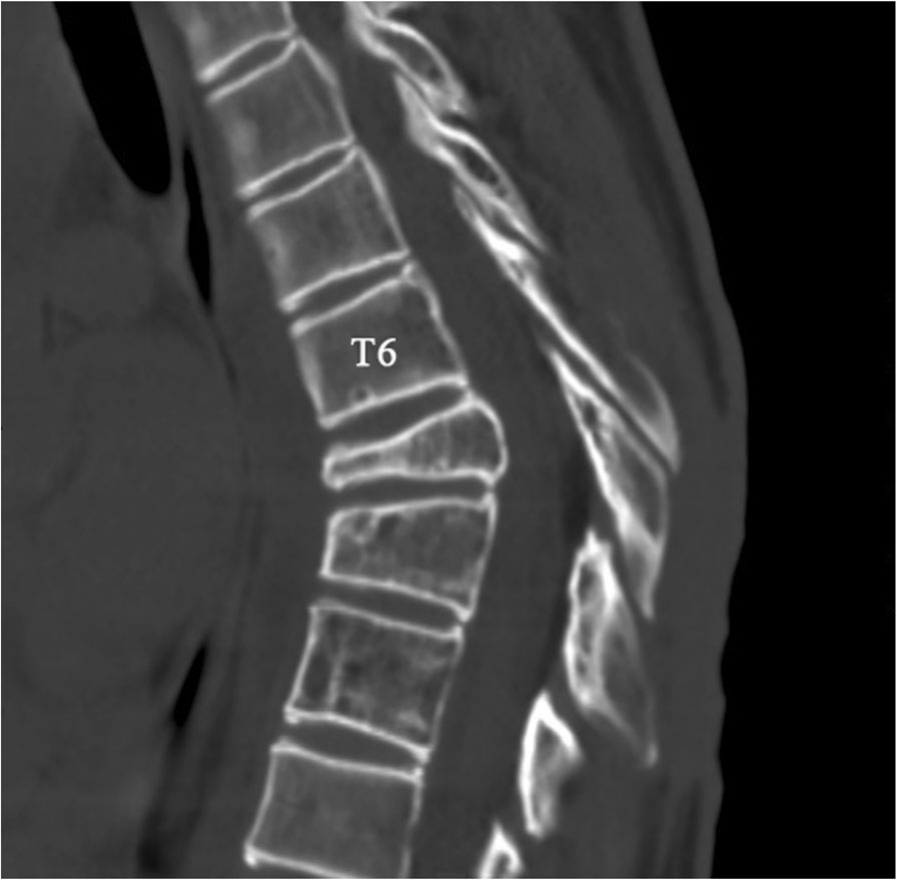
Thoracic CT at 2-year follow-up. Axial and sagittal CT scans show bilateral pleural thickening and minimal pleural effusion; changes in the thoracic spine and osteolytic rib stopped. (The white arrow indicates the destructed vertebrae, the red arrow indicates the osteolytic rib, and the circle indicates pleural effusion)

**Fig. 11:**
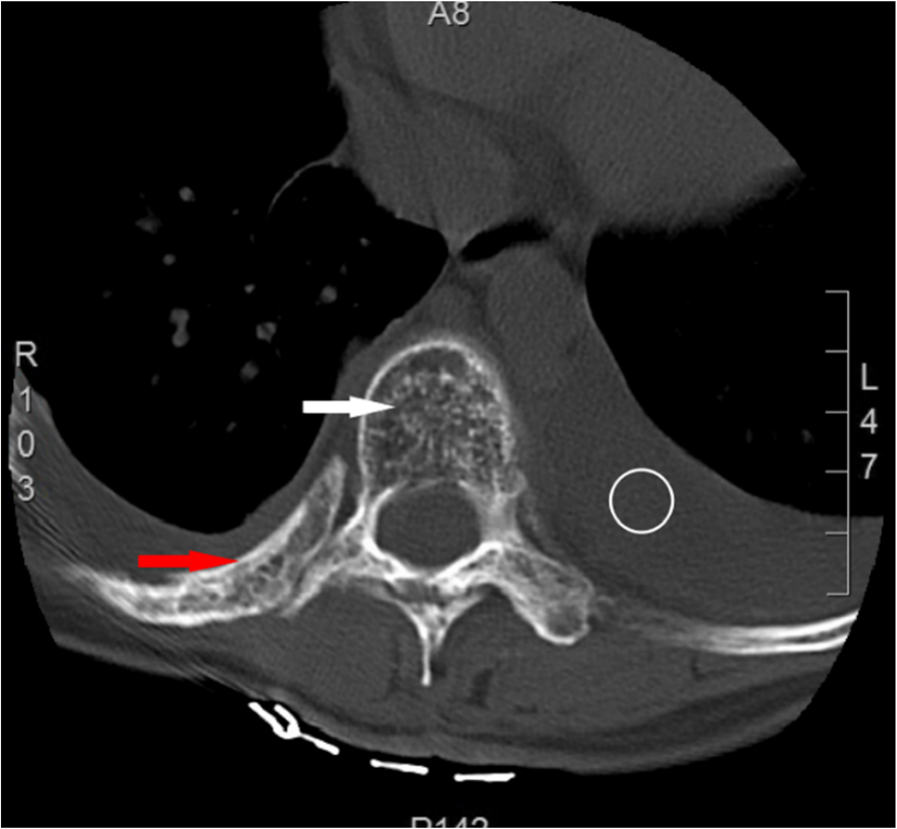
Thoracic CT at 2-year follow-up. Axial and sagittal CT scans show bilateral pleural thickening and minimal pleural effusion; changes in the thoracic spine and osteolytic rib stopped. (The white arrow indicates the destructed vertebrae, the red arrow indicates the osteolytic rib, and the circle indicates pleural effusion)

No treatment has been approved for GSS; thus, several treatment methods are used. In our case, the treatment plan was discussed and confirmed in a multidisciplinary clinic meeting. Bisphosphonates and vitamin D therapy were administered to treat the disease because the patient was vitamin D deficient, and the disease is self-limiting. Because the neurological exam showed no abnormalities, conservative treatment was considered, and a custom-made polypropylene body jacket was prescribed to prevent kyphotic deformity. Her clinical status improved steadily following the oral bisphosphonates and vitamin D supplementation. A final thoracic CT (Figs. 10 and 11) was performed 2 years after diagnosis and showed a successful reduction in the amount of pleural fluid and stabilization of the thoracic spine deformity.

## Discussion

GSS is also known as disappearing bone disease, massive osteolysis, and vanishing bone disease. Gorham and Stout tabulated 24 previously reported cases and presented the disease as a syndrome in 1954 [5]. The disease’s etiology and pathogenesis are unclear. Dellinger et al. [6] discussed the potential roles of endothelial cells, osteoclasts and osteoblasts in the GSS pathogenesis. They thought that lymphatic endothelial cells secreted factors and influenced osteoclast and/or osteoblast activities, thus leading to skeletal disorders. Some researchers also think that minor traumas increase osteoclast activity and lead to osteolysis. However, the molecular mechanisms and genetic bases that drive the osteolysis and lymphangiogenesis in GSS are unclear. GSS presents no familial association or sex preference and can occur at any age, but it is more common in adolescents and young adults [7]. GSS may involve any part of the skeleton; however, the skull, shoulder and pelvis are the most commonly involved sites. Primary involvement of the spine is less common and has only been described in approximately 50 cases [8]. Chylothorax occurs when this disease invades the pleura or thoracic duct, and several reports describe an association between GSS and chylothorax [9, 10] Bilateral chylothorax is usually fatal and may lead to progressive respiratory failure, with a mortality rate increase to 53% [11].

The clinical features of GSS vary and depend on the site of involvement and time of diagnosis. Common symptoms include pain, weakness, limited motion, and spontaneous pathological fractures [12]. GSS is difficult to diagnose, particularly in the early stage. It is often misdiagnosed as a neoplasm, tuberculosis or chronic osteomyelitis due to its rarity and unique clinical characteristics [13]. Heffez et al. [14] suggested exclusive diagnostic criteria in 1983, which must be based on combined clinical, radiological, and histopathological findings. Radiographs provide the most significant clues for reaching a diagnosis. The radiological appearance of bone lesions varies and depends on the stage. Plain radiographs show osteolysis or pathological fractures. CT scanning and three-dimensional reconstruction are useful for accurately assessing the range of bone destruction. MRI is useful for ascertaining disease extension and soft tissue involvement [15]. Histopathological examination is the gold standard. GSS pathology shows no evidence of malignant, neuropathic, or infectious components involved in causation, except for lymphovascular malformations in the bone [16]. Our patient’s biopsy revealed that the lesion was composed of hyperplastic blood vessels and fibrous tissues, which is consistent with the pathological features of GSS.

No standard therapy is available for GSS. Several treatments have been proposed, including medications, surgical intervention, radiotherapy and/or a combination of these [17]. Prognosis depends on the site of involvement, extent of the disease and presence of complications. Chylothorax is likely the most severe complication and can lead to death. Managing chylothorax in GSS includes irradiation therapies, pleurectomy, pleurodesis, thoracic duct ligation and lymphangiomatous tissue excision [2]. Suero et al. [18] recommended pleurodesis for intractable pleural effusion. In our case, chest drainage and thoracic duct ligation combined with pleurodesis were performed to relieve the symptoms due to the refractory chylothorax. Progressive bilateral chylothorax after thoracic duct ligation may be due to thoracic duct invasion or communication between the lymphatic dysplasia and the pleural cavity. In our patient, ligating the right thoracic duct increased the pressure inside the left duct, leading to pleural effusion in the left hemithorax.

Spinal lesions may be managed via radiation therapy, braces, balloon vertebroplasty, vertebral osteotomy or debridement and bone graft fusion to maintain stability and prevent neurological injury [19–21]. Because the disease is often self-limiting, if a patient has no severe deformity or progressive neurologic deficits, it may be better to prioritize the use of conservative treatments [22]. In our case, low-dose radiotherapy was suggested to prevent osteolytic progression, but we chose to combine an antiosteoclastic medication with other conservative modalities after considering the adverse side effects of radiotherapy. Bisphosphonates and vitamin D were administered for 2 years, and the final CT showed no clear osteolytic progression.

## Conclusions

This report describes a case of GSS affecting the thoracic spine with progressive bilateral chylothorax, which was treated successfully. GSS is a rare disorder of the musculoskeletal system that responds poorly to therapies and has high morbidity and mortality. Chylothorax is a common complication, and physicians should be vigilant about possible serious pulmonary complications when lesions involve the thoracic spine and make appropriate management decisions. A review of the literature as well as the results of the present case support the need for further studies to elucidate the pathogenesis of GSS with chylothorax and seek effective therapies.

## Abbreviations

CT: Computed tomography
GSS: Gorham-Stout syndrome
MRI: Magnetic resonance imaging
